# Klf4-Tymp axis promotes inflammation-driven early tumorigenesis by enhancing kras mutation-induced acinar-to-ductal metaplasia through Pi3k/Akt and Mek/Erk pathways

**DOI:** 10.1186/s13046-026-03712-8

**Published:** 2026-05-07

**Authors:** Qihang Yuan, Junhong Chen, Peng Luo, Kai Liu, Fangyue Guo, Yao Xu, Mengying Tong, Hong Xiang, Dong Shang

**Affiliations:** 1 Pancreas and Biliary Center, Department of General Surgery, First Affiliated Hospital of Dalian Medical University, Dalian, Liaoning China; 2 Clinical Laboratory of Integrative Medicine, First Affiliated Hospital of Dalian Medical University, Dalian, Liaoning China; 3 Department of Hepatobiliary and Pancreatic Surgery, General Surgery Center, First Hospital of Jilin University, Changchun, Jilin China; 4 Department of Oncology, Zhujiang Hospital, Southern Medical University, Guangzhou, China; 5 Department of Ultrasound, First Affiliated Hospital of Dalian Medical University, Dalian, China; 6 Institute (College) of Integrative Medicine, Dalian Medical University, Dalian, Liaoning China

**Keywords:** ScRNA-seq, KLF4, TYMP, Acinar-to-Ductal Metaplasia, Inflammation, Pancreatic Tumorigenesis

## Abstract

**Background:**

Acinar-to-ductal metaplasia (ADM) is a pivotal step in pancreatic tumorigenesis, reversible in normal contexts but progressing to PanIN and pancreatic cancer (PC) in the presence of Kras mutation and inflammation. Thus, delineating exocrine cell heterogeneity and identifying regulators of ADM are essential for understanding pancreatic tumorigenesis.

**Methods:**

We collected single-cell RNA sequencing (scRNA-seq) data of 146 human pancreatic samples, followed by comprehensive exploration of dynamic change and heterogeneity of ADM via machine learning algorithms. Multi-omics analysis integrating ATAC-seq and RNA-seq highlights the association of Klf4-Tymp axis with ADM. A dual-luciferase reporter assay was performed to evaluate the transcriptional activation of the Tymp promoter by Klf4. AAV-mediated gene silencing was performed in vivo to elucidate the roles and underlying mechanisms of the Klf4/Tymp axis during the ADM process in Pdx1-Cre; Kras^G12D/+^ (KC) mice. In-vitro mouse pancreatic organoid model combined with amino acid mutation was established to investigate the role of Tymp in regulating ADM and its underlying mechanisms.

**Results:**

scRNA-seq revealed one S100A4^+^ acinar subpopulation and six ductal subpopulations (GPX1^+^, CD24^+^, TFF3^+^, MT-ATP8^+^, S100A9^+^, and HMGB2^+^) associated with poor prognosis in PC. Integrated pseudotime analysis of pancreatic acinar and ductal cells highlighted the potential involvement of TYMP in regulating the pathological process of ADM. Klf4 was identified as an upstream transcription factor regulating the Tymp gene. ATAC-seq revealed increased chromatin accessibility at the Klf4 and Tymp locus under inflammatory injury or oncogenic Kras conditions. Dual-luciferase reporter assays demonstrated that the transcription factor Klf4 binds to the promoter region of Tymp, thereby promoting its transcriptional expression. Pancreatic overexpression of Klf4 in mice upregulated Tymp expression and facilitated inflammation-induced initiation and progression of ADM. Exogenous supplementation with Tipiracil Hydrochloride suppressed the effects induced by Klf4. Knockdown of the Tymp gene suppressed inflammation-driven initiation and progression of ADM in KC mice through Pi3k/Akt and Mek/Erk pathways.

**Conclusions:**

The Klf4-Tymp axis promotes inflammation-associated early pancreatic tumorigenesis by enhancing KRAS mutation-induced ADM through activation of the Pi3k/Akt and Mek/Erk signaling pathways. These findings provide new insights into the molecular mechanisms linking inflammatory injury to ADM and early pancreatic tumorigenesis.

**Supplementary Information:**

The online version contains supplementary material available at 10.1186/s13046-026-03712-8.

## Introduction

Pancreatic cancer (PC) is among the most lethal malignancies, with a dismal 5-year survival rate around 12% despite modern treatments [1]. However, if the disease is detected at an early stage, the 5-year survival rate can increase to 44% [2]. The majority of PCs arise through a stepwise progression beginning with acinar-to-ductal metaplasia (ADM). ADM can be triggered by pancreatic injury (e.g. chronic pancreatitis) or by oncogenic KRAS signaling [3]. Importantly, oncogenic KRAS is found in over 90% of human PanIN lesions and PCs [4], and KRAS mutation in mouse acinar is sufficient to induce ADM and low-grade PanIN formation. Under sustained KRAS activation and inflammation stimulation, ADM becomes persistent, rather than transient, and predisposes tissue to neoplastic transformation [5]. ADM provides a regenerative response to injury, but it is also a fertile ground for tumor initiation. Despite its key role, the specific molecular factors that allow ADM cells to survive, proliferate and ultimately progress toward malignancy remain incompletely understood. Elucidating these early drivers of inflammation-induced tumorigenesis is critical, since they may represent targets for intervention or chemoprevention in high-risk settings.

Single-cell RNA sequencing (scRNA-seq) has recently enabled unprecedented resolution of cellular heterogeneity in the pancreas during ADM and tumor initiation [6]. By profiling thousands of individual cells, scRNA-seq can resolve distinct epithelial subpopulations and capture dynamic transitions that are invisible to bulk RNA analysis [6]. For example, Schlesinger et al. [7] performed scRNA-seq on pancreas from a Kras-driven mouse model spanning preinvasive lesions to carcinoma. They identified metaplastic cells derived from acinar cells that express the transcription factors Onecut2 and Foxq1, and further defined six distinct types and states of acinar metaplastic cells. These metaplastic cells coexisted with immune and stromal cells that contribute to an immunosuppressive microenvironment supporting tumor growth. Similarly, recent scRNA-seq of human PC patient tumors revealed diverse epithelial clusters [8]. Researchers found that MMP1 played a key role in the transition of normal pancreatic cells to cancer cells. Knockdown of MMP1 reduced the expression of ductal markers such as SOX9, and vice versa. Together, these findings highlight that scRNA-seq can identify rare or transient states during ADM and early tumorigenesis, and can nominate candidate regulators (e.g. Onecut2, Foxq1) of the metaplastic process. However, existing single-cell studies have mostly been descriptive. In particular, a comprehensive single-cell atlas of acinar reprogramming in vivo, especially one incorporating targeted perturbations of candidate factors, is still lacking.

Among the many factors implicated in epithelial plasticity and inflammation, kruppel-like factor 4 (Klf4) and thymidine phosphorylase (Tymp) stand out but have not been studied together in ADM. Klf4 is a zinc-finger transcription factor well known for roles in differentiation, epithelial barrier function, and stemness [9]. Wei et al. [10] showed that Klf4 is upregulated by Kras mutation or injury and is required for acinar-to-ductal reprogramming. Klf4 deletion in Kras mutation mice almost completely blocked PanIN formation, whereas ectopic Klf4 expression in acinar cells suppressed acinar markers and induced ductal markers. A recent review of KLFs in pancreatic physiology similarly notes that KLF4 is upregulated during ADM and PanIN, and that its deletion attenuates Kras-driven neoplasia [11]. Tymp (also known as PD-ECGF) is an enzyme in nucleotide salvage that has emerged as a pro-tumorigenic factor in many contexts [12]. Besides its metabolic role, Tymp strongly promotes angiogenesis, inflammation, and cell survival [13]. For example, Tymp enhances tumor growth by stimulating angiogenic factor release and by activating anti-apoptotic and epigenetic pathways [14]. Yang et al. [15] showed that Tymp was frequently overexpressed in cancers and drived cell proliferation, epithelial-mesenchymal transition (EMT) and invasiveness. Despite these broad effects, Tymp’s role in the normal or inflamed pancreas is unexplored. No studies have examined whether Tymp is activated during ADM or whether it collaborates with transcriptional factors like Klf4.

In this study, we integrated publicly available single-cell RNA sequencing data from 146 human pancreatic samples. By subsetting acinar and ductal cell populations, we performed subtype classification, functional annotation, and prognostic analysis. During the late acinar-to-early ductal transition, two genes, KLF5 and TYMP, emerged as particularly prominent regulators. While KLF5 has been extensively reported as a driver of ADM in the pancreas [16, 17], the role of TYMP in this process has not previously been elucidated. Through transcription factor prediction analysis, we identified KLF4 as a potential upstream regulator of TYMP expression. Based on these findings, we proposed the hypothesis that the Klf4-Tymp axis represents a novel mechanism that enhances inflammation-induced the occurrence and progression of ADM in KC mouse (PDX1-Cre; Kras^G12D/+^) mice. Our results demonstrated that the KLF4-TYMP axis promotes pancreatic ADM by regulating the PI3K and MAPK pathways, thereby facilitating the initiation of early pancreatic tumorigenesis.

## Methods

### Chemicals and reagents

Caerulein was purchased from MeilunBio (Dalian, China). Adeno-associated virus (AAV) targeting Tymp and Klf4 were obtained from OBiO Technology Co., Ltd. (Shanghai, China). The TYMP-targeting shRNAs were designed and synthesized by GenePharma Technology Co., Ltd. (Suzhou, China). All primer used for PCR experiments were designed and synthesized by Sangon Biotech (Shanghai, China). Organoid culture medium was purchased from bioGenous Technology Co., Ltd. (Shanghai, China). PI3K activator (740Y-P), Tymp inhibitor (Tipiracil hydrochloride), MEK inhibitor (PD98059) was purchased from MedChemExpress. Human pancreatic cancer tissue microarray was purchased from Weiao Technology Co., Ltd. (Shanghai, China). Lipofectamine™ 3000 Transfection Reagent was purchased from Thermo Fisher Scientific (Waltham, USA). Wild-type and mutant Tymp plasmids were designed and synthesized by Zolgene Biotechnology Co., Ltd (Fuzhou, China). Dual luciferase reporter gene assay kit was provided by CWBIO Technology Co., Ltd. (Taizhou, China).

### Animal experiments

All animal experiments were reviewed and approved by the Institutional Animal Care and Use Committee of Dalian Medical University and First Hospital of Jilin University. Male C57BL/6 mice (6–8 weeks old) were purchased from Beijing HFK Bioscience Co., Ltd. (Beijing, China) and acclimated for one week prior to experimentation. A reversible acinar-to-ductal metaplasia (ADM) model was established by intraperitoneal injection of caerulein (100 µg/kg per injection, eight injections per day at 1-h intervals, for two consecutive days). Pancreatic tissues were harvested at baseline (before injection) and on days 1, 2, and 14 post-injection. Male KC mice (6–8 weeks old, Cyagen, China) were used to model irreversible ADM under inflammation conditions. Caerulein was administered intraperitoneally at 50 µg/kg per injection, four injections per day at 1-h intervals, three times per week (Monday, Wednesday, Friday) for two consecutive weeks before tissue collection. For gene silencing studies, mice received intraperitoneal injection of AAV, and experiments were performed 2–3 weeks later to allow sufficient viral transduction in the pancreas.

### Single-cell RNA sequencing analysis

A total of 146 human pancreatic samples were collected from publicly available single-cell RNA sequencing datasets (CRA001160, GSE154778, GSE155698, GSE165045, GSE197177, GSE205049, GSE212966, GSE214295, and GSE242230). A total of 97 tumor samples, 10 metastatic samples, 30 normal pancreatic samples, and 9 chronic pancreatitis samples were analyzed. Raw data were processed using the R language, including quality control, removal of doublets, and batch correction. Cell type annotation was performed based on canonical markers. Dimensionality reduction and visualization were carried out using Seurat and plot1cell packages, while marker heatmaps were generated with the scRNAtoolVis package. Inflammatory signaling within cells was evaluated using the AddModuleScore algorithm.

For exocrine cell heterogeneity analysis, ductal epithelial and acinar cells were re-clustered and subjected to secondary dimensionality reduction. ClusterGVis and org.Hs.eg.db packages were applied to generate marker-based heatmaps and functional enrichment analyses. Functional pathway variation between sub-clusters was assessed using the GSVA package with gene sets from msigdbr. Cell fate trajectories were inferred using Monocle2, Monocle3, Slingshot, and CytoTRACE. Differentially expressed genes associated with the acinar-to-ductal transition were identified using the FindMarkers function.

### ATAC-seq and WGBS-seq analysis

The GSE132330 dataset provides ATAC-seq data of pancreatic injury and Kras-induced ADM. After obtaining the bw files, we imported them into IGV for visualization. The GSE249140 cohort provides WGBS-seq sequencing results of doxycycline-induced Klf4 expression in mouse pancreas. Mouse genome annotation was performed based on the TxDb.Mmusculus.UCSC.mm10.knownGene package, followed by methylation data processing. Finally, visualization was achieved using the ggplot2 package.

### RNA-seq analysis

First, AAV technology was employed to specifically knock down Tymp expression in the pancreas of KC mice. After confirmation of AAV-mediated gene silencing, caerulein was administered intraperitoneally to accelerate pancreatic injury-induced ADM progression. Pancreatic tissues were collected and subjected to RNA sequencing, which was performed by Shanghai Applied Protein Technology Co., Ltd. (Shanghai, China). Following sequencing, the TPM-formatted data were systematically analyzed, including differential expression analysis and pathway enrichment analysis.

### Cell culture

The murine pancreatic cancer cell line Panc02 was obtained from Procell Life Science & Technology Co., Ltd. (Wuhan, China) and cultured in DMEM medium supplemented with 10% fetal bovine serum (FBS) and 1% penicillin-streptomycin under standard conditions (37 °C, 5% CO₂). Primary pancreatic cells were isolated and cultured according to protocols described in our previous publications [18, 19]. H6C7, PANC-1, and SW1990 cell lines were purchased from Procell Life Science & Technology Co., Ltd. (Wuhan, China) and cultured in DMEM medium.

### Cell transfection and cell counting kit-8 (CCK8)

The Tymp-shRNAs were transfected according to the instructions of the Lipo3000 transfection reagent manual, and the knockdown efficiency was verified by PCR. The detailed shRNA sequences were as follows: shTYMP#1 (5’-CTTGTGGACAAGCATTCCACA-3’), shTYMP#2 (5’-GCTGGAGTCTATTCCTGGATT-3’), shTYMP#3 (5’-CAGCCTCCATTCTCAGTAAGA-3’). The two shRNA sequences with the most prominent knockdown efficiency were selected for subsequent experiments. The CCK8 kit was purchased from MeilunBio (Dalian, China), and the experiment was conducted following the manufacturer’s instructions. Briefly, the successfully knockdown PANC1 cells were seeded in a 96-well plate at an appropriate density. At 24, 48, and 72 h, 10% CCK8 solution was added to the wells for detection.

### Wound-healing and transwell invasion assays

PANC1 cells were transfected using the Lipo3000 transfection reagent kit in a six-well plate. Once the cell density reached an appropriate level, a 200 µL pipette tip was used to create a scratch. Images were captured under a microscope at 0 and 24 h after scratching. The invasion assay was performed using a transwell chamber (NEST Biotechnology, Wuxi, China). Briefly, after successful transfection, cells were seeded at an appropriate density onto the pre-coated transwell chamber with matrigel (Mogengel Bio, Xiamen, China). A serum concentration gradient was set to induce the cells to migrate through the matrigel to the underside of the transwell chamber. After 24 h, images were taken under a microscope to observe the differences in the number of invading cells.

### Quantitative real-time PCR

Total RNA was extracted using 1 mL TRIzol reagent (Adamas Life, China) according to the manufacturer’s instructions. Briefly, cells were lysed and incubated for 5–10 min at room temperature, followed by the addition of 200 µL chloroform. After vigorous mixing and centrifugation at 12,000 g for 15 min, the aqueous phase was collected, mixed with 500 µL isopropanol, and centrifuged again. The RNA pellet was washed with pre-chilled 80% ethanol, air-dried, and dissolved in RNase-free water. RNA concentration and purity were measured spectrophotometrically. Complementary DNA (cDNA) was synthesized using the All-in-One First-Strand Synthesis MasterMix (Yugong Life Technology Co., Ltd., China). Quantitative real-time PCR (qPCR) was performed using Taq-HS SYBR^®^ Green qPCR Premix (Yugong Life Technology Co., Ltd., China) on a real-time PCR detection system (Bioer Technology Co., Ltd., China). Reaction mixtures were prepared according to the manufacturer’s instructions. Primer sequences are listed in Supplementary Table 1.

### Western blot analysis

Pancreatic tissues were immediately snap-frozen in liquid nitrogen for 30 min after dissection and stored at − 80 °C until protein extraction. Tissues were homogenized with lysis buffer supplemented with phosphatase inhibitors, protease inhibitors, and PMSF using a tissue grinder with sterile beads for 2–3 min. Lysates were incubated on ice for 15–20 min with intermittent vortexing (30s every 5 min), followed by centrifugation at 12,000 g for 15 min at 4 °C. Supernatants were collected, quantified using a BCA assay, and denatured by boiling in loading buffer at 100 °C for 10 min. Appropriate amounts of protein were separated on precast SDS-PAGE gels (Yeasen Biotechnology, China; or Servicebio Technology, China) and transferred to PVDF membranes using a rapid transfer solution (Servicebio Technology, China). Membranes were blocked with rapid blocking buffer (Beyotime Biotechnology, China) for 15 min at room temperature and incubated overnight at 4 °C with primary antibodies at appropriate dilutions. After three washes with TBST, membranes were incubated with HRP-conjugated secondary antibodies for 30–60 min at room temperature, followed by three additional washes. Protein bands were visualized using Tanon™ High-sig ECL detection reagent. Antibodies used in this study are listed in Supplementary Table 2.

### Hematoxylin and Eosin (HE) staining

HE staining was performed on paraffin-embedded tissue sections using a commercial Hematoxylin-Eosin Stain Kit (Cat. G1120, Solarbio, China) according to the manufacturer’s instructions. Stained sections were mounted with neutral resin and imaged under a light microscope.

### Immunohistochemistry (IHC)

Paraffin-embedded tissue sections were dewaxed with environmentally friendly dewaxing solution and rehydrated through a graded ethanol series. Antigen retrieval was performed using an appropriate retrieval buffer, followed by quenching of endogenous peroxidase activity with 3% hydrogen peroxide. Sections were blocked with BSA and incubated with primary antibodies overnight at 4 °C. On the following day, slides were incubated with species-appropriate secondary antibodies, followed by visualization with DAB substrate. Counterstaining was performed with hematoxylin, including differentiation and bluing steps. Finally, sections were dehydrated through graded ethanol, cleared in xylene, and mounted. Detailed information on the primary antibodies used in this study is provided in Supplementary Table 2.

### Immunofluorescence (IF)

Immunofluorescence double staining for amylase and CK19 was performed on paraffin-embedded pancreatic sections. After deparaffinization, rehydration, and heat-induced antigen retrieval (30 min), sections were washed in PBS and blocked with 3% BSA. Slides were incubated overnight at 4 °C with mouse anti-CK19 (1:500, Servicebio, GB12197) and rabbit anti-amylase (1:200, Protech, 12540-1-AP), then exposed to Alexa Fluor 488-conjugated donkey anti-mouse IgG and Cy3-conjugated donkey anti-rabbit IgG (50 min, room temperature, light-protected). Nuclei were counterstained with DAPI, and samples were mounted with antifade medium. Images were captured using an Olympus fluorescence microscope with appropriate filter settings (DAPI, Alexa Fluor 488, Cy3).

### Sirius red and alcian blue staining

Both sirius red and alcian blue staining kits were purchased from Servicebio Technology Co., Ltd. (Wuhan, China). The staining process was performed according to the manufacturer’s instructions.

### Pancreatic organoid culture

Murine pancreatic tissues were finely minced in a dish containing digestion buffer composed of collagenase type I and trypsin inhibitor. Samples were incubated at 37 °C with 5% CO₂ for 20–25 min, after which digestion was terminated. The suspension was centrifuged, and the pellet was treated with red blood cell lysis buffer for 2–4 min, followed by centrifugation and multiple washes with HBSS. The final cell pellet was resuspended in DMEM medium supplemented with matrigel and seeded into six-well plates to allow organoid formation. Organoids were subsequently maintained in a commercial organoid culture medium under standard conditions.

### Dual-luciferase reporter gene assay

Klf4 overexpression plasmid (1 µg), luciferase reporter plasmid (1 µg), and RL-TK plasmid (0.2 µg) were transfected using Lipo3000 reagent. Twenty-four hours post-transfection, the assay was performed according to the instructions of the Dual Luciferase Reporter Gene Assay Kit. Briefly, cells were first lysed using Passive Lysis Buffer, while firefly and renilla luciferase assay reagents were prepared. Chemiluminescence was then measured using a multimode microplate reader (BioTek Instruments, Inc.).

### Statistical analysis

Comparisons between two groups were performed using Student’s t-test or the Wilcoxon rank-sum test, as appropriate. For comparisons among three or more groups, one-way analysis of variance (ANOVA) or the Kruskal-Wallis test was applied. The crystal structure of P19971 protein and the 3D structures of thymidine and Tipiracil (TPI) were used for molecular docking with AutoDock Vina, after preparation and conversion to the required format. The top-scoring docking conformation was visualized using PyMol. Data visualization and statistical analyses were conducted using GraphPad Prism and R software. A p-value < 0.05 was considered statistically significant.

## Results

### Integrated single-cell landscape of human pancreatic samples

**Fig. 1:**
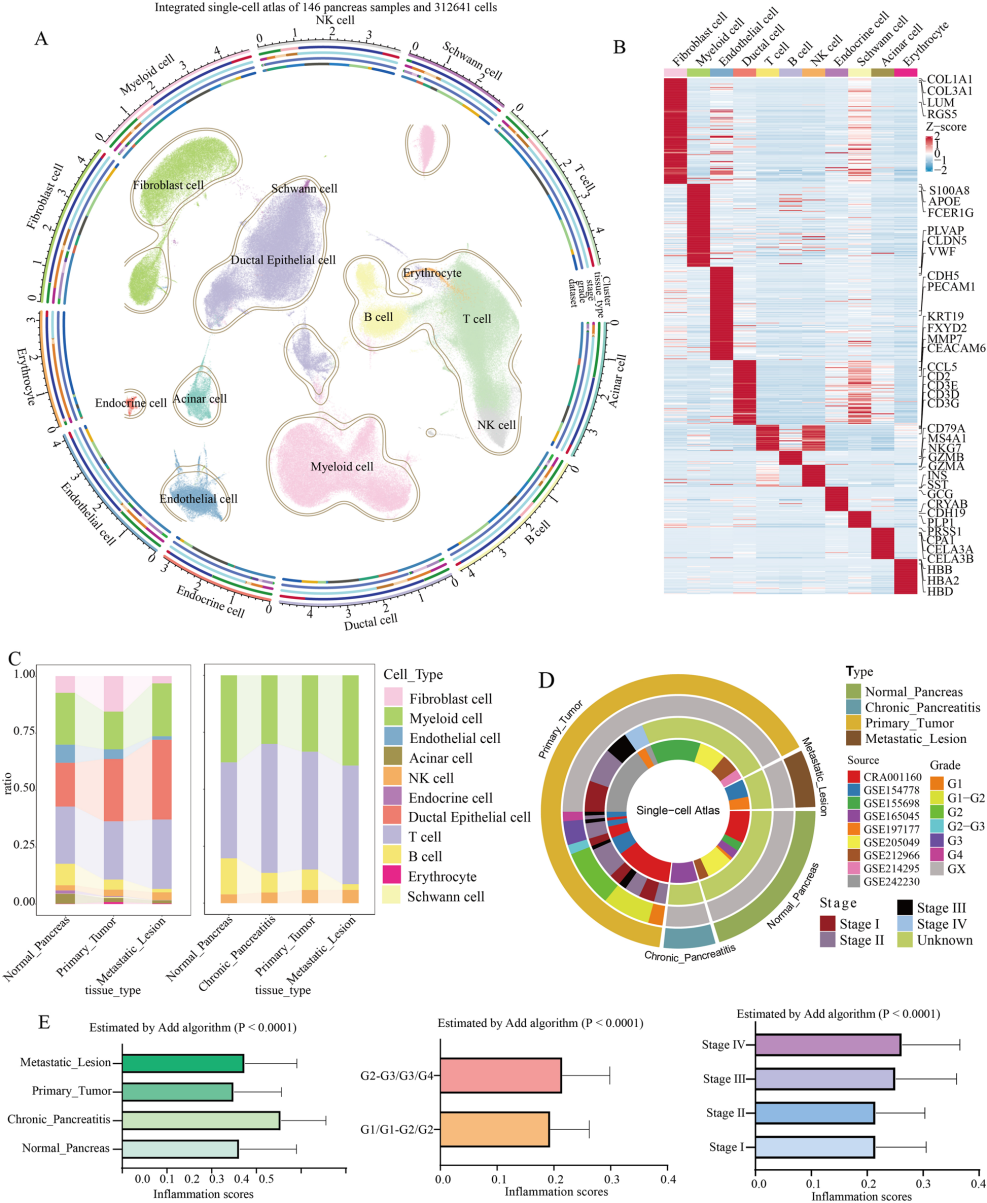
Integrated single-cell atlas of the human pancreas across normal, chronic pancreatitis, primary tumor, and metastatic states. **A** The UMAP plot of 312,641 cells from 146 pancreatic samples showing major cell populations. **B** The heatmap of representative marker genes delineating distinct pancreatic cell types. **C** Proportional distributions of cell types across normal pancreas, chronic pancreatitis, primary tumors, and metastatic lesions. **D** The circular plot integrating sample sources and clinical traits. **E** Quantitative analysis of inflammation scores across disease states (left), pathological grades (middle), and tumor stages (right)

To construct a comprehensive atlas of the human pancreas, we integrated single-cell RNA-seq datasets from 146 samples, encompassing 312,641 cells. Dimensionality reduction and clustering revealed a broad cellular heterogeneity across the pancreas (Fig. 1A). Distinct clusters were identified, including 75,718 ductal epithelial cells, 6632 acinar cells, 35,977 fibroblasts, 13,842 endothelial cells, 61,021 myeloid cells, 88,803 T cells, 17,197 B cells, 9131 NK cells, 981 Schwann cells, 1766 erythrocytes, and 1573 endocrine cells, providing a high-resolution map of the pancreatic microenvironment. Cell type annotation was validated by canonical marker expression, as illustrated by the heatmap of representative genes (Fig. 1B). For example, COL1A1 and COL1A2 characterized fibroblasts, VWF and PECAM1 defined endothelial cells, and KRT19 identified ductal epithelial cells. These results confirmed the reliability of clustering and annotation.

We next compared the cellular composition among normal pancreas, chronic pancreatitis, primary tumor, and metastatic lesions (Fig. 1C). Notably, primary tumors were enriched for ductal epithelial cells and fibroblasts, while acinar cells and endothelial cells were markedly reduced compared with normal pancreas. Chronic pancreatitis samples displayed an intermediate cellular composition, suggesting progressive remodeling during disease evolution. The 146 integrated samples were further stratified by clinical parameters, including data source, disease type, tumor stage, and grade (Fig. 1D).

Finally, we evaluated inflammatory signaling using the Add algorithm (Fig. 1E). Inflammation scores were significantly elevated in chronic pancreatitis compared with normal pancreas, primary tumors and metastatic lesions (*P* < 0.0001). Moreover, higher inflammation scores were associated with advanced pathological grades and late clinical stages, highlighting the close link between inflammatory signaling and disease progression.

### Secondary clustering of ductal epithelial cells reveals intrinsic heterogeneity

**Fig. 2:**
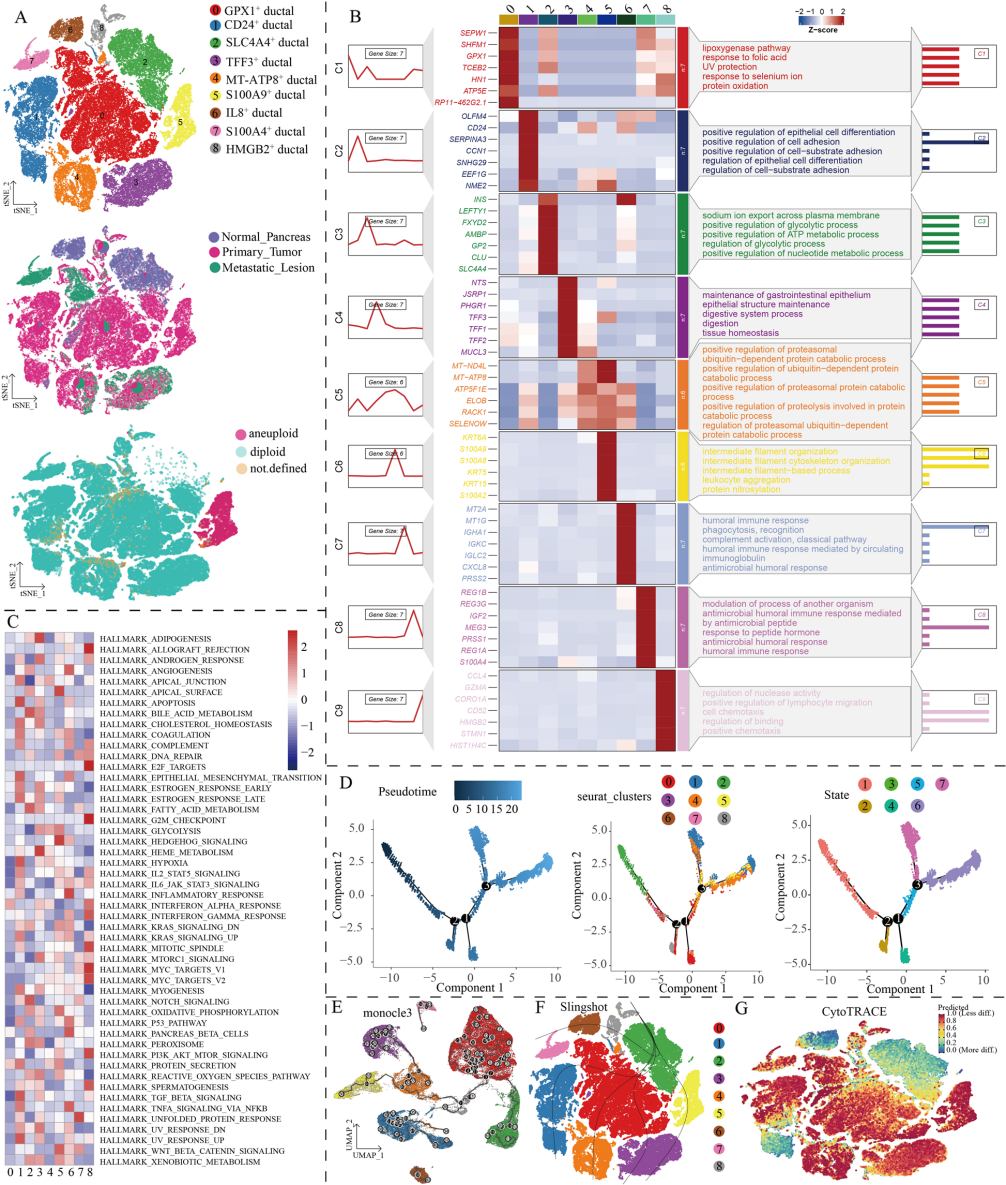
Single-cell transcriptomic analysis reveals heterogeneity of ductal cell subpopulations. **A** t-SNE plots showing the distribution of ductal cell subclusters identified by marker gene expression. **B** Heatmap of representative marker genes and functional enrichment analyses. **C** Hallmark pathway scores for each ductal subcluster demonstrating functional diversity across cell states. Pseudotime trajectory analysis reveals dynamic lineage progression among ductal cell subtypes. Trajectories were inferred using (**D**) Monocle2, (**E**) Monocle3, (**F**) Slingshot, and (**G**) CytoTRACE algorithms

To further investigate the heterogeneity of ductal epithelial cells, we performed secondary clustering analysis after their extraction from the integrated data. The t-SNE plot revealed nine distinct ductal subclusters (Fig. 2A). Normal pancreatic tissue was mainly composed of ductal cells from clusters 2 and 6, while the ductal cells in primary tumors primarily originated from clusters 0, 1, 2, 3, 4, 5, and 8. In addition, analysis of copy number variation distinguished diploid, aneuploid, and undefined states, indicating that cluster 5 ductal cells were the predominant source of malignant ductal cells.

Marker gene expression and pathway enrichment analyses demonstrated unique molecular signatures for each subcluster (Fig. 2B). For example, Cluster 0 exhibited high expression of GPX1, suggesting a link to the lipoxygenase pathway. In contrast, Cluster 2 was defined by SLC4A4 expression and was enriched for genes associated with glycolytic process. After identifying the marker genes for each ductal cell subtype, prognostic analysis was performed using TCGA data. The results showed that, except for ductal subpopulations 2, 6, and 7, all other ductal cell types were associated with poor prognosis (Supplementary Fig. 1).

To further delineate the biological functions of each ductal subtypes, we performed hallmark pathway enrichment analysis (Fig. 2C). Distinct transcriptional programs were observed among the subclusters, highlighting divergent functional states. Clusters enriched for glycolysis, oxidative phosphorylation, fatty acid metabolism, and bile acid metabolism suggested marked differences in metabolic reprogramming across ductal cells. Notably, the S100A9⁺ ductal cluster (Cluster 5) exhibited enhanced glycolytic and Wnt/β-catenin pathways, consistent with a pro-tumorigenic phenotype. In contrast, the SLC4A4⁺ (Cluster 2) and IL8⁺ (Cluster 6) clusters displayed enrichment in pathways related to pancreas β cells and angiogenesis. Several clusters demonstrated activation of cancer-associated signaling pathways, including KRAS, MYC, PI3K/AKT/mTOR, TGF-β, Notch, Hedgehog, and p53 signaling, underscoring their potential involvement in occurrence and development of tumors. In addition, immune and inflammatory pathways such as IL2-STAT5, IL6-JAK-STAT3, TNF-α/NF-κB signaling, and interferon responses, were variably enriched, suggesting differential immunoregulatory roles across the ductal subsets. Together, these findings indicate that ductal cell subclusters are not only metabolically distinct but also differentially engage oncogenic and immune-related programs, providing important clues to their potential contributions to pancreatic tumorigenesis.

Using the monocle2 algorithm, we performed pseudotime analysis and identified a branched differentiation trajectory. In this trajectory, Cluster 2 was positioned at the root state, suggesting that it may represent an early progenitor-like population (Fig. 2D). The trajectory branched into multiple terminal states, and Clusters 1, 4, and 5 were identified as potential differentiated endpoints. This distribution suggests that ductal cells follow divergent lineage programs, likely corresponding to distinct functional fates. To validate the robustness of these findings, we applied alternative trajectory inference algorithms, including Monocle3, Slingshot, and CytoTRACE (Figs. 2E-G). All methods consistently supported the presence of Cluster 2 as the starting point and confirmed Clusters 1, 4, and 5 as terminal branches along the trajectory. Moreover, CytoTRACE analysis further highlighted reduced differentiation potential in these terminal clusters compared with the root state, reinforcing their role as more mature or specialized ductal subsets. Together, these results indicate that ductal epithelial cells undergo heterogeneous lineage progression, with distinct clusters occupying progenitor, intermediate, and terminal states. This trajectory framework provides mechanistic insights into how ductal cell heterogeneity may contribute to pancreatic disease evolution and malignant transformation.

#### Heterogeneity of acinar cells and lineage transition toward ductal states

**Fig. 3:**
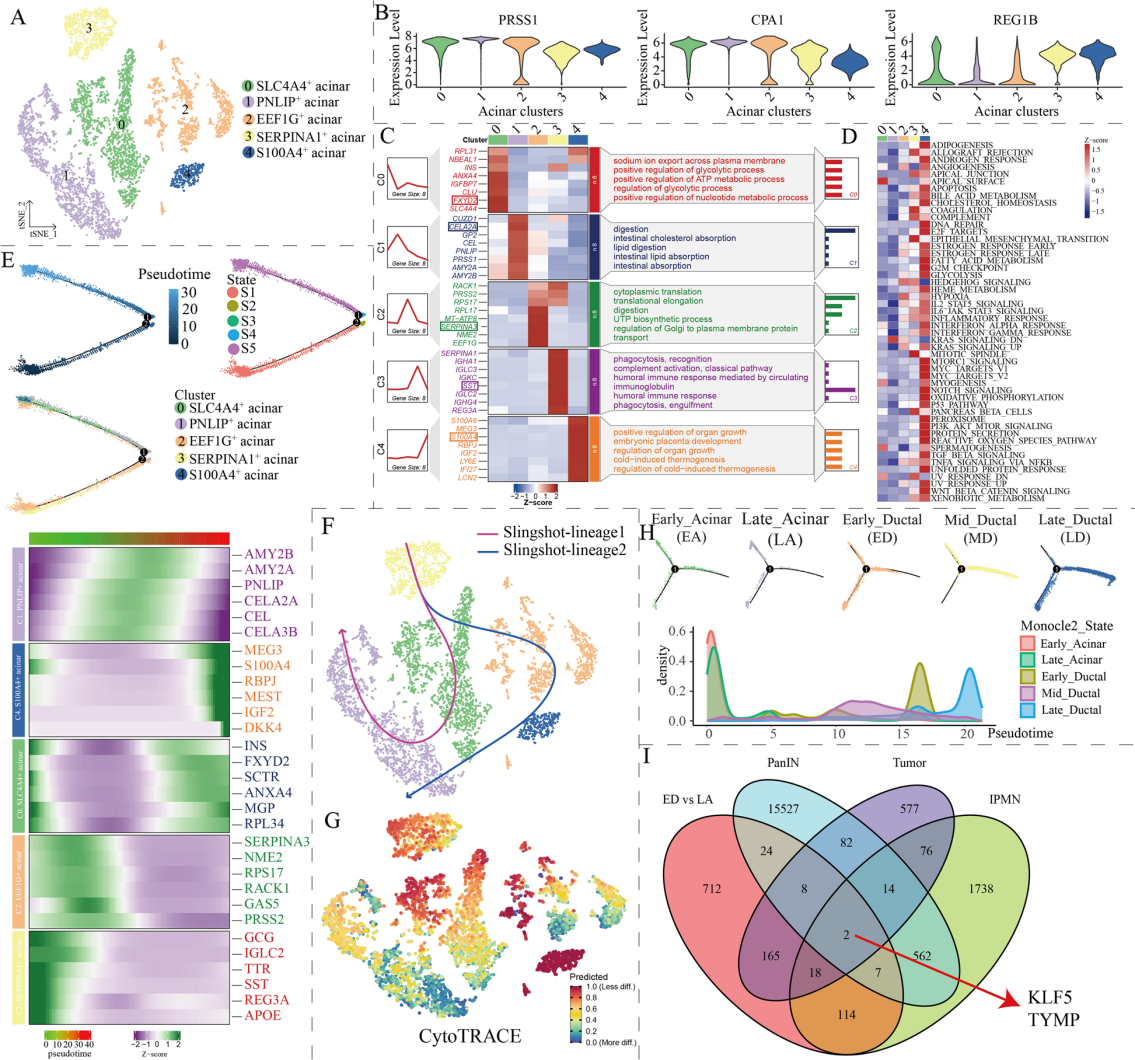
Acinar cell heterogeneity and lineage trajectories underlying acinar-to-ductal metaplasia (ADM). **A** UMAP projection of acinar cells showing distinct transcriptional clusters defined by representative markers (SLC4A4⁺, PNLIP⁺, EEF1G⁺, SERPINA1⁺, and S100A4⁺). **B** Violin plots displaying expression of canonical acinar markers (PRSS1, CPA1, and REG1B) across clusters. **C** Heatmap with functional enrichment analysis of acinar subclusters’ markers. **D** Hallmark gene set scores distinguishing biological programs across acinar subclusters. Pseudotime trajectory analysis mapping the sequential transition from early to late acinar states based on (**E**) Monocle2 algorithm, (**F**) Slingshot algorithm and (**G**) CytoTRACE algorithm. **H** Monocle2 trajectory analysis delineating five major cell states across the ADM continuum, including early acinar (EA), late acinar (LA), early ductal (ED), mid ductal (MD), and late ductal (LD) populations. **I** Venn diagram highlights the hub genes (i.e. KLF5 and TYMP) associated with tumor occurrence through comparing differentially expressed genes between ED versus LA states, PanIN, IPMN and tumor datasets

After completion of the analysis of ductal epithelial cells, we next focused on acinar cells, the other major exocrine cell type in the pancreas. Secondary clustering revealed five distinct acinar subclusters (Fig. 3A). Despite this heterogeneity, all clusters consistently expressed classical acinar markers, including PRSS1, CPA1, and REG1B, confirming the robustness of acinar cell identification (Fig. 3B).

Functional characterization through marker heatmaps, pathway enrichment, and hallmark gene set analysis further highlighted distinct molecular features among the acinar subsets (Figs. 3C-D). For instance, specific clusters were enriched in pathways related to digestion and cholesterol absorption, while others showed signatures of immune interaction and metabolic regulation, underscoring the functional diversity of acinar cells. Similar to the analysis strategy applied to the ductal subpopulations, we screened the marker genes of each acinar cell subtype and performed prognostic analysis based on TCGA data. We found that S100A4^+^ acinar cells were associated with poor prognosis in pancreatic cancer (Supplementary Fig. 2).

To investigate acinar cell plasticity, we performed pseudotime trajectory analysis using the monocle2 algorithm. This analysis placed the SERPINA1^+^ acinar cluster at the root of the trajectory, representing an early progenitor-like state, while the S100A4^+^ acinar cluster occupied the terminal state, suggesting a transdifferentiated phenotype (Fig. 3E). Notably, EEF1G^+^ acinar cells were distributed throughout the trajectory, indicating their persistence across developmental stages. The trajectory structure was validated by additional algorithms, including Slingshot and CytoTRACE (Figs. 3F-G).

By integrating acinar and ductal cells, we reconstructed a continuous ADM spectrum that could be stratified into five distinct states: early acinar (EA), late acinar (LA), early ductal (ED), mid ductal (MD), and late ductal (LD) (Fig. 3H). This framework recapitulates the gradual phenotypic remodeling during ADM, bridging normal acinar physiology and ductal-like phenotypes associated with neoplastic progression. To pinpoint conserved regulators of ADM and pancreatic tumorigenesis, we performed cross-dataset comparisons, intersecting differentially expressed genes between ED and LA states with genes from PanIN progression, primary tumor versus normal pancreas, and IPMN lesions. This integrative analysis highlighted a small set of shared genes, among which KLF5 and TYMP emerged as strong candidates (Fig. 3I).

Subsequently, we also integrated acinar and ductal cells to explore the expression of KLF5 and TYMP in PRSS1^+^KRT19^+^ cells. As shown in Supplementary Fig. 3 A and 3B, PRSS1^+^ cells and KRT19^+^ cells demonstrated obvious differences. PRSS1^+^ cells were characterized by high expression of PRSS1 and CPA1, while KRT19^+^ cells were characterized by high expression of KRT19 and SOX9 (Supplementary Fig. 3 C and D). Compared with PRSS1^+^ cells, the expression of KLF5 and TYMP were significantly higher in PRSS1^+^KRT19^+^ cells (Supplementary Fig. 3E and F), suggesting their pivotal roles in driving acinar-to-ductal transition and early pancreatic neoplasia. Previous studies [16, 17] have reported that KLF5 promotes the formation of pancreatic ADM; however, the role of TYMP has not yet been described. Therefore, the subsequent experiments in this study were designed to investigate the function of TYMP.

Using the BEST online platform, we analyzed the relationship between TYMP expression and clinical parameters of pancreatic cancer. Our results showed that TYMP was markedly overexpressed in pancreatic cancer tissues compared with normal pancreatic tissues (Supplementary Fig. 4 A), and its expression was correlated with tumor stage (Supplementary Fig. 4B) and grade (Supplementary Fig. 4 C). Patients with high TYMP expression exhibited poorer prognosis (Supplementary Fig. 4D). In addition, compared with normal pancreatic epithelial cells (H6C7), pancreatic cancer cell lines (PANC1 and SW1990) displayed higher TYMP expression levels (Supplementary Fig. 4E). Immunohistochemical analysis based on pancreatic cancer tissue microarrays further confirmed that TYMP protein expression was also elevated in tumor tissues (Supplementary Fig. 4 F).

### TYMP expression is elevated during ADM and enhanced by kras mutation

**Fig. 4:**
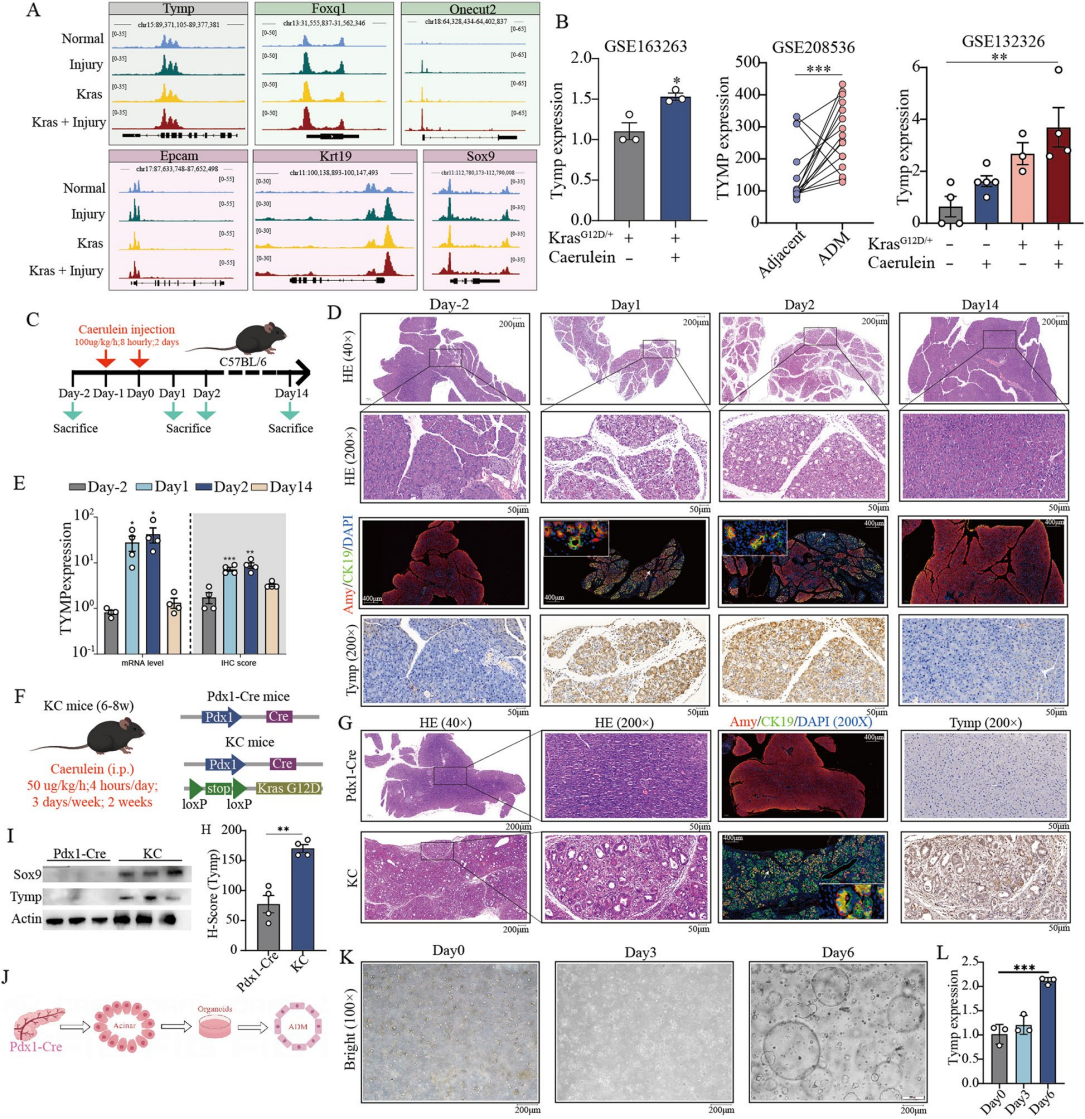
TYMP expression is upregulated during acinar-to-ductal metaplasia (ADM). **A** ATAC-seq analysis of pancreatic tissues from normal, caerulein-injured wild-type (WT), Kras-mutant, and caerulein-treated Kras-mutant mice. **B** Public transcriptome datasets (GSE163263, GSE208536, and GSE132326) demonstrating elevated TYMP expression in ADM. **C** Schematic of caerulein-induced reversible ADM in C57BL/6 mice. **D** Representative HE, immunofluorescence (Amy/CK19/DAPI), and immunohistochemistry (TYMP) images across indicated time points (Day 1, Day 2, Day 14). **E** Quantification of TYMP mRNA and protein expression. **F** Schematic of caerulein-induced irreversible ADM in KC mice. **G** HE, immunofluorescence (Amy/CK19/DAPI), and IHC (Tymp) showing consistent upregulation of TYMP in KC-driven ADM. **H** Quantitative IHC analysis of Tymp expression. **I** Pancreatic tissues from Pdx1-Cre and KC mice analyzed by Western blot for Sox9 and Tymp. **J** Mouse pancreatic organoid cultures derived from Pdx1-Cre mice. **K** Representative bright-field images (Day 0, Day 3, Day 6) and (**L**) qPCR quantification showing a progressive increase in TYMP expression during organoid-derived ADM

To validate the role of TYMP in ADM, we first examined chromatin accessibility and transcriptomic changes from public datasets. As shown in Fig. 4A, loci of Tymp, Foxq1, Onecut2, Epcam, Krt19, and Sox9 exhibited increased accessibility under injury conditions, and this effect was further amplified in the presence of Kras^G12D^ mutation, suggesting a synergistic regulation of ductal reprogramming-related genes. Consistently, re-analysis of multiple independent datasets (GSE163263, GSE208536, and GSE132326) confirmed a significant upregulation of TYMP expression during ADM in both mouse and human pancreatic tissues (Fig. 4B). To experimentally verify these observations, caerulein-induced reversible ADM models were established in C57BL/6 mice (Fig. 4C). HE staining on days 1 and 2 post-modeling clearly showed evidence of ADM (Fig. 4D). However, by day 14, ADM lesions had disappeared and the cells had re-differentiated into acinar cells. Subsequently, we confirmed the occurrence of ADM on days 1 and 2 through immunofluorescence staining for amylase and CK19 (Fig. 4D). Interestingly, we also found that Tymp expression levels were elevated on days 1 and 2 post-modeling but returned to normal levels by day 14 (Fig. 4D and E). These findings suggested that Tymp might be involved in the process of reversible ADM in the pancreas. Additionally, caerulein-induced irreversible ADM models were also established in KC mice (Pdx1-Cre; Kras^G12D/+^) (Fig. 4F). Both IHC (Fig. 4G-H) and WB (Fig. 4I) demonstrated markedly elevated Tymp expression in KC mice, in parallel with enhanced Sox9 expression and histological evidence of ADM formation. Immunofluorescence staining for Amylase/CK19 confirmed ductal transdifferentiation (Fig. 4G), and these findings further support the role of Tymp in Kras-driven ADM.

In addition to the in vivo experiments, we employed an organoid-based in vitro model to recapitulate the process of ADM (Fig. 4J). Over time, acinar cells progressively transdifferentiated into duct-like cells, a transition that was accompanied by a marked increase in Tymp gene expression (Fig. 4K and L). Together, these results established TYMP as a dynamically regulated gene during ADM. Its expression was induced by pancreatic injury, further elevated in the context of Kras mutation, and strongly associated with ADM formation and progression toward ductal lesions. These findings provide mechanistic evidence linking TYMP to early pancreatic tumorigenesis.

#### In vivo validation of AAV-mediated infection efficiency and systemic toxicity

First, we performed Tymp knockdown experiments in Panc02 cells and primary pancreatic acinar cells (Supplementary Fig. 5 A and 5B), and found that the shRNA corresponding to the sh-355 sequence exhibited the most efficient knockdown performance. Therefore, we selected this sequence to construct the AAV vector. To evaluate the efficiency of AAV-mediated Tymp knockdown, we administered escalating doses of AAV-shRNA(Tymp) or control vectors into mice. Ex Vivo Organ Imaging in Small Animals demonstrated dose-dependent viral transduction in pancreatic tissue, with robust reporter signals observed at higher doses (≥ 6 × 10^11vg/animal) (Supplementary Fig. 6 A). To evaluate the pancreatic infection efficiency of the AAV8 vector, we utilized the intrinsic Gdgreen reporter carried by the virus. Fluorescence imaging revealed robust green signals within the pancreas across all AAV8-shRNA treatment groups, with intensity correlating to the viral dosage (Supplementary Fig. 6B). These findings confirmed efficient AAV-mediated delivery into pancreatic tissue. Additionally, we found that the AAV vector used in this study had minimal impact on Tymp gene expression in mouse liver (Supplementary Fig. 7). To assess the biosafety of AAV-mediated gene silencing, we measured serum biochemical parameters, including ALT, AST, ALB, BUN, and CREA. All values remained within the physiological range, with no significant differences between experimental and control groups, indicating minimal systemic toxicity (Supplementary Fig. 6 C). HE staining revealed preserved pancreatic architecture in all groups, without evidence of inflammation, necrosis, or fibrosis (Supplementary Fig. 6D). These findings confirmed that AAV8-mediated delivery effectively silenced Tymp in the pancreas without causing obvious tissue damage or systemic toxicity.

#### In vivo knockdown of TYMP suppresses ADM progression and fibrotic remodeling

**Fig. 5:**
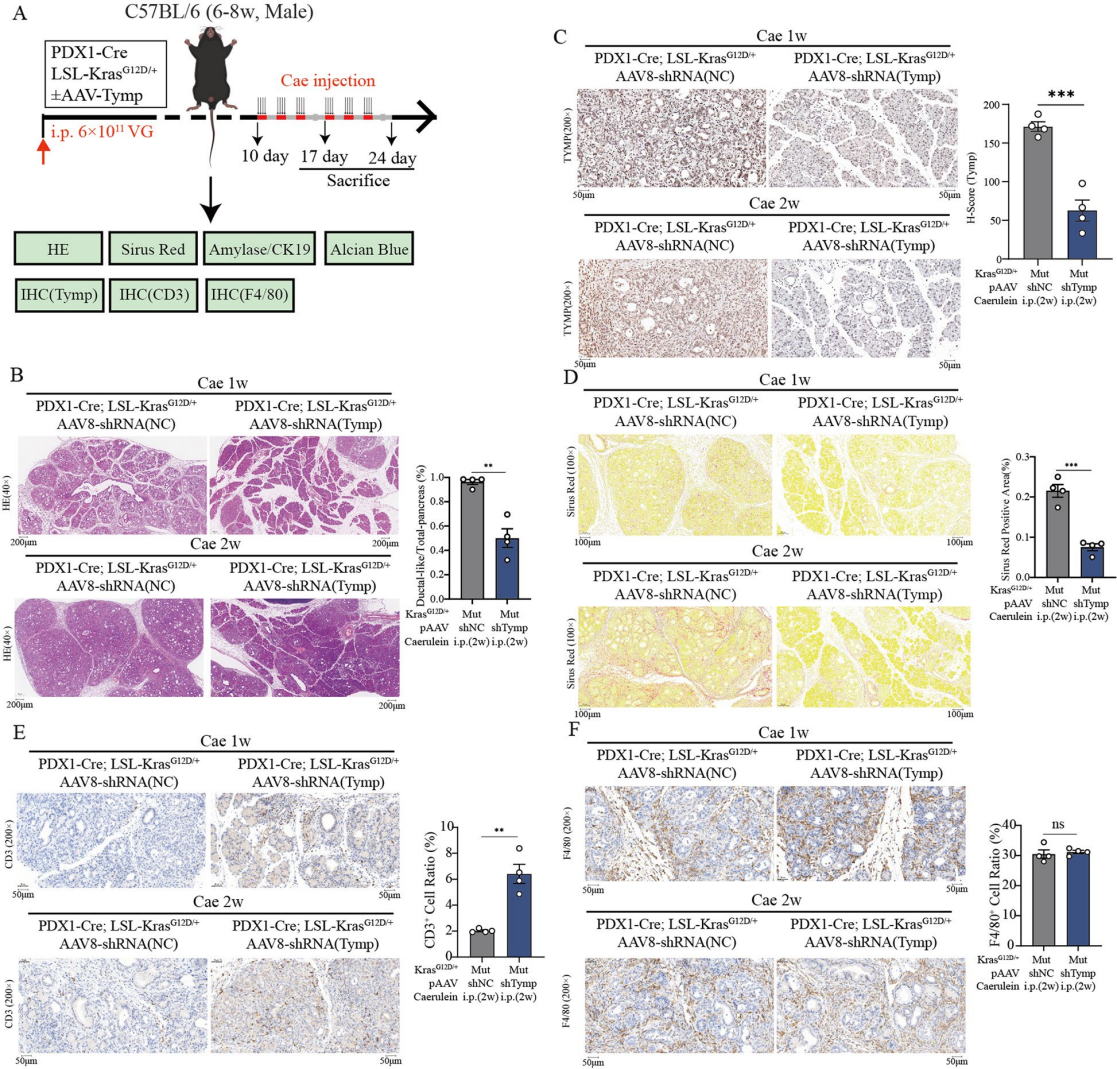
Tymp knockdown suppresses ADM formation in KC mice. **A** Schematic diagram of the animal modeling procedure. AAV was administered intraperitoneally into KC mice at a dose of 6 × 10¹¹vg. Ten days later, the mice were subjected to a two-week caerulein treatment protocol, followed by assessment of relevant pathological indicators. **B** HE images, (**C**) Tymp immunohistochemistry images, (**D**) Sirius red staining, (**E**) CD3 immunohistochemistry images, and (**F**) F4/80 immunohistochemistry images of pancreatic tissues from KC mice with control or Tymp knockdown. (**, *p* < 0.01; ***, *p* < 0.001; ns, no significance)

To validate the functional role of Tymp during ADM, we performed in vivo knockdown experiments using an AAV8-shRNA delivery system in KC mice (Fig. 5A). Following viral transduction, caerulein was administered intraperitoneally for two consecutive weeks to accelerate pancreatic injury. Histological examination demonstrated that control mice developed extensive ADM lesions, whereas Tymp knockdown markedly reduced ductal-like acinar structures, as observed at both 1 and 2 weeks (Fig. 5B). Immunohistochemistry revealed a significant reduction of Tymp protein expression in AAV-shTymp-treated KC mice compared with controls (Fig. 5C). Moreover, sirius red (Fig. 5D) staining demonstrated diminished collagen deposition, indicating that Tymp silencing alleviated fibrosis associated with ADM. These results suggest that Tymp not only regulates epithelial plasticity but also contributes to stromal remodeling during pancreatic injury. Alcian blue assays (Supplementary Fig. 8A) indicated that after two weeks of caerulein stimulation, the control group exhibited a certain proportion of PanIN-like structures, whereas such pathological features were rarely observed in the Tymp knockdown group. Immunofluorescence staining for amylase and CK19 (Supplementary Fig. 8B) further confirmed that Tymp knockdown suppressed the emergence of ductal-like lesions in KC mice. Considering that the immune microenvironment, particularly T cells and macrophages, was critical for pancreatic tumorigenesis, we further investigated the impact of Tymp knockdown on the infiltration of T cells and macrophages. As shown in Figs. 5E and F, Tymp knockdown resulted in a relative reduction in the proportion of CD3^+^ cells, while no significant change was observed in the proportion of F4/80^+^ cells.

#### Knockdown of TYMP suppresses proliferation, migration, and invasion of pancreatic cancer cells

The PCR experiment confirmed that shTYMP #1 and #2 sequences exhibited efficient knockdown efficiency in PANC1 cells (Supplementary Fig. 9 A). Therefore, these two sequences were selected to investigate the effect of TYMP on tumor cell proliferation, migration, and invasion. CCK8 assays showed that silencing TYMP inhibited the proliferation of PANC1 cells at 48 h and 72 h, but not at 24 h (Supplementary Fig. 9B-D). Wound healing assays (Supplementary Fig. 9E) and transwell assays (Supplementary Fig. 9 F) demonstrated that TYMP knockdown suppressed the migration and invasion of PANC1 cells.

### Klf4 is identified as a potential upstream regulator of Tymp

**Fig. 6:**
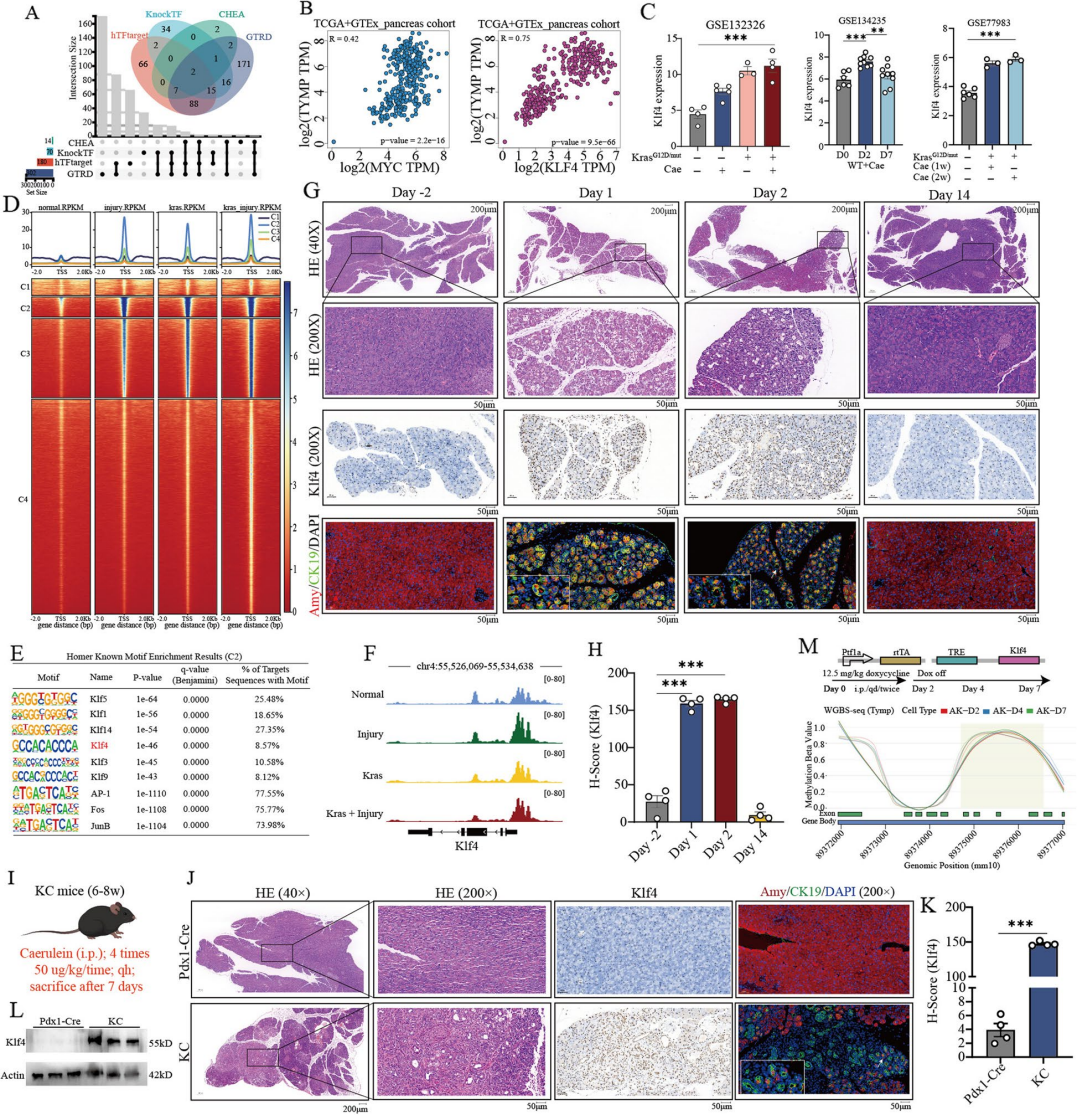
Klf4 acts as an upstream regulator of Tymp during ADM. **A** Transcription factor prediction analysis across multiple databases (KnockTF, CHEA, GTRD, hTFtarget) identifying MYC and KLF4 as candidate regulators of TYMP. **B** Correlation analysis using TCGA and GTEx datasets showing significant positive associations of TYMP with MYC and KLF4. **C** Public transcriptome datasets (GSE132326, GSE134325, GSE77983) showing Klf4 upregulation during caerulein-induced ADM and in Kras-mutant models. **D** ATAC-seq of pancreatic tissues from normal, caerulein-injured WT, Kras-mutant, and Kras-mutant with injury groups, highlighting increased chromatin accessibility at C2 peaks under ADM conditions. **E** Motif enrichment analysis of C2 peaks revealing enrichment of Klf family binding motifs. **F** Genome browser tracks showing enhanced chromatin accessibility at the Klf4 locus in Kras-mutant and injury conditions. **G** Histological assays showing dynamic upregulation of Klf4 at early stages (Day 1–2) and reduction at Day 14. **H** Quantitative analysis of Klf4 immunohistochemistry in reversible ADM model. **I** Schematic of caerulein-induced irreversible ADM in KC mice. **J** Representative HE, immunohistochemistry, and immunofluorescence in KC mice showing persistent Klf4 expression in irreversible ADM. **K** Quantitative analysis of Klf4 immunohistochemistry in irreversible ADM model. **L** Western blot assays confirming elevated Klf4 protein expression in KC pancreas compared with controls. **M** Whole-genome bisulfite sequencing (WGBS) showing peak Klf4 expression at Day 2 and progressive reduction by Day 7, accompanied by increased DNA methylation at the Tymp locus (chr15: 89,375,000–89,376,000)

To identify upstream transcriptional regulators of Tymp, we first performed transcription factor prediction across multiple databases. By integrating intersecting candidates, both MYC and KLF4 were highlighted as potential transcriptional regulators of TYMP (Fig. 6A). Correlation analysis using the GEPIA2 platform further confirmed that TYMP expression was strongly associated with both MYC and KLF4 across pancreas datasets (Fig. 6B). Consistently, public transcriptome data revealed that Klf4 expression levels were positively correlated with the occurrence of ADM, suggesting its potential involvement in ADM reprogramming (Fig. 6C). The chromatin accessibility profiles demonstrated distinct clustering patterns, among which the C2 peak set showed markedly increased accessibility under both pancreatic injury and Kras mutation (Fig. 6D). Motif enrichment analysis of the C2 peaks revealed significant enrichment of the Klf family, with Klf4 being particularly prominent (Fig. 6E). Genome browser views confirmed that the chromatin accessibility at the Klf4 locus was markedly enhanced in conditions of pancreatic injury and Kras mutation (Fig. 6F). In WT mice with caerulein-induced reversible ADM, Klf4 expression increased at the onset of ADM but diminished as ADM regressed, paralleling the transient nature of the process (Fig. 6G and H). In contrast, Klf4 expression remained elevated in KC mice, in which ADM is irreversible, supporting a sustained role for Klf4 in neoplastic progression (Fig. 6I-K). Western blot assays further confirmed the upregulation of Klf4 protein in pancreatic tissues from KC mice compared to controls (Fig. 6L).

To validate whether Klf4 directly regulates Tymp transcription, we analyzed the promoter region of the Tymp gene and identified two putative Klf4 binding sites located within − 2000 bp upstream of the transcription start site (TSS) (Supplementary Fig. 10 A). Site-directed mutagenesis was used to generate two mutant constructs (MUT1 and MUT2) (Supplementary Fig. 10 A). Dual-luciferase reporter assays demonstrated that overexpression of Klf4 significantly increased the activity of the wild-type Tymp promoter compared with the control vector (*p* < 0.01). Mutation of the Klf4 binding site (MUT1 and MUT2) markedly abolished Klf4-mediated activation of the Tymp promoter (*p* < 0.01) (Supplementary Fig. 10B). To investigate the regulatory effect of Klf4 on Tymp expression and its role in ADM in vivo, we overexpressed Klf4 in the pancreatic tissues of KC mice using AAV vectors. Twenty-one days later, the modeling procedure was initiated as illustrated in Supplementary Fig. 10C. AAV-mediated overexpression of Klf4 in the pancreas of KC mice aggravated caerulein-induced ADM progression, accompanied by increased Tymp expression (Supplementary Fig. 10D). Subsequently, to further explore whether Tymp was a key effector molecule mediating the function of Klf4, we established an in vitro organoid model (Supplementary Fig. 10E). TPI, a classical inhibitor of Tymp, suppressed Klf4 overexpression-induced ADM formation in vitro, as evidenced by a marked reduction in the size of duct-like structures (Supplementary Fig. 10 F). These findings indicated that Klf4 directly bound to and activated the Tymp promoter through specific motifs, thereby enhancing its transcriptional activity.

Finally, WGBS analysis revealed temporal epigenetic dynamics associated with Klf4 expression. Klf4 expression peaked at day 2 following doxycycline treatment, but progressively declined from day 4 to day 7. Strikingly, as Klf4 levels decreased, the DNA methylation within the TYMP genomic region (chr15: 89,375,000–89,376,000) gradually increased (Fig. 6M). These results suggested that Klf4 acted to suppress DNA methylation at the Tymp locus, thereby maintaining chromatin accessibility and promoting Tymp transcription.

### Tymp knockdown suppresses ADM processing of KC mice through Pi3k/Akt and Mek/Erk Signaling Pathways

**Fig. 7:**
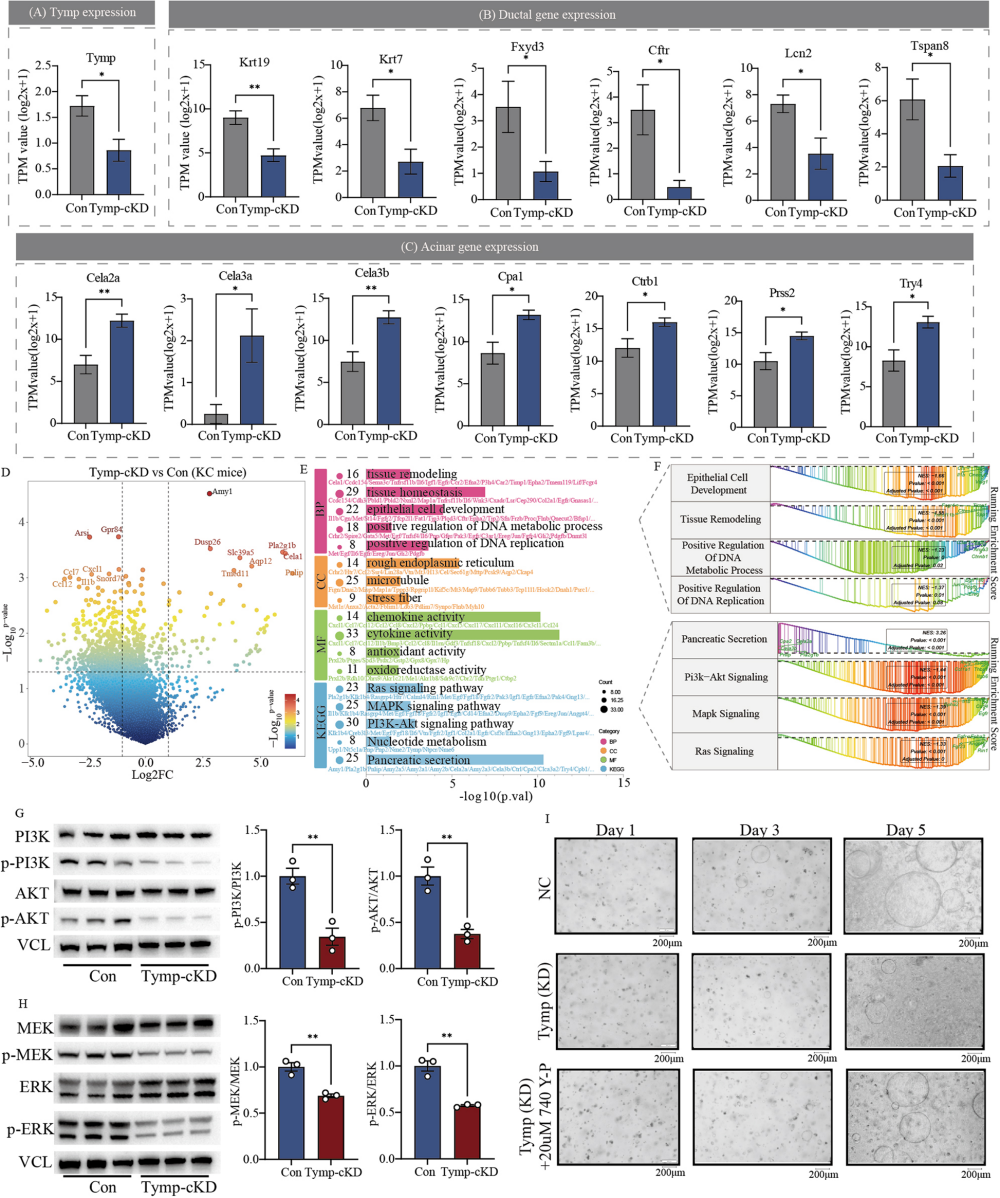
Tymp knockdown suppresses ADM processing of KC mice through Pi3k/Akt and Mek/Erk signaling pathways. **A** Validation of Tymp knockdown efficiency by RNA-seq in KC mice. **B** Expression of ductal-associated genes (Krt19, Krt7, Fxyd3, Ctr, Lcn2, and Tspan8) showing significant reduction following Tymp knockdown. **C** Expression of acinar-associated genes (Cela2a, Cela3a, Cela3b, Cpa1, Ctrb1, Prss2, and Try4) showing restoration upon Tymp knockdown. **D** Volcano plot of differentially expressed genes in Tymp-knockdown versus control KC mice. **E** GO and KEGG analysis of downregulated genes highlighting alterations in epithelial cell development, DNA replication, and key signaling pathways. **F** GSEA analysis. **G** Western blot assays showing reduced phosphorylation of PI3K and AKT in Tymp-knockdown KC pancreas. **H** Western blot assays showing reduced phosphorylation of MEK and ERK in Tymp-knockdown KC pancreas. **I** Effect of 740 Y-P on ADM in an in vitro organoid model following Tymp knockdown

RNA sequencing of pancreatic tissues from KC mice with AAV-mediated Tymp knockdown revealed marked transcriptional reprogramming. Tymp expression was significantly reduced in the knockdown group, confirming effective silencing (Fig. 7A). Ductal markers (Krt19, Krt7, Fxyd3, Cfr, Lcn2, Tspan8) were consistently downregulated, while acinar markers (Cela2a, Cela3a, Cela3b, Cpa1, Ctrb1, Prss2, Try4) were significantly upregulated (Fig. 7B-C). These results suggest that Tymp inhibition blocks ADM and promotes acinar lineage maintenance.

Volcano plot and enrichment analyses demonstrated broad transcriptional changes after Tymp depletion (Fig. 7D-E). Pathway analysis revealed downregulation of epithelial development, tissue remodeling, and DNA replication programs, alongside suppression of oncogenic signaling cascades including Ras, Pi3k/Akt, and Mek/Erk pathways. Conversely, pathways related to pancreatic secretion and digestive enzyme production were enriched (Fig. 7F). Western blot assays corroborated the transcriptomic findings by showing reduced phosphorylation of PI3K and AKT, as well as MEK and ERK, in Tymp-knockdown tissues (Fig. 7G-H). Total protein levels of PI3K, AKT, MEK, and ERK remained unchanged, indicating pathway-specific inhibition. 740 Y-P, an agonist of the PI3K pathway, reversed the Tymp knockdown-induced inhibition of ADM in an in vitro organoid model (Fig. 7I and Supplementary Fig. 11B). In other words, while Tymp knockdown suppressed ADM formation in the organoid model, exogenous supplementation with 740 Y-P rescued this phenotype. Similarly, we found that exogenous supplementation with PD98059 suppressed the enhancement of ADM induced by Tymp overexpression (Supplementary Fig. 11 A and C).

To further investigate whether Tymp’s ADM induction depend on its enzymatic activity, we constructed Tymp mutants. As shown in Supplementary Figs. 12 A and B, the amino acid sequences of human TYMP and mouse Tymp proteins are highly conserved. Molecular docking results elucidated several key amino acid residues that constitute the primary region responsible for its enzymatic activity (Supplementary Figs. 12 C and D). After amino acid sequence alignment, we found that these critical residues differ by approximately 14 amino acids between the human and mouse sequences. Previous studies [20, 21] have reported that mutations H116F, Y199A, R202E, S217A, and K221E effectively inhibit the enzymatic activity of human TYMP. Therefore, after mapping to the corresponding positions in the mouse sequence, we generated a similar mutant construct containing all five amino acid substitutions. As shown in Supplementary Fig. 12E, ADM induction by the Tymp mutant in vitro was substantially weaker than that induced by wild-type Tymp, indicating that Tymp-mediated regulation of ADM was dependent on its enzymatic activity.

## Discussion

The scRNA-seq has emerged as a powerful tool to profile complex tissues like the pancreas at single-cell resolution, enabling identification of diverse cell types and states within both normal and diseased pancreatic tissues [22]. In PC and its precursor lesions, scRNA-seq approaches have provided in-depth characterization of cellular heterogeneity and plasticity that was previously unappreciated with bulk analyses [23, 24]. By dissecting the transcriptomes of individual cells, scRNA-seq can resolve rare or transient cell populations and delineate lineage relationships, thereby shedding light on the progression from healthy pancreas to pre-neoplastic lesions and invasive cancer [7]. Our study provides a comprehensive single-cell atlas of the human pancreas across healthy and diseased states, revealing unprecedented detail about cellular heterogeneity and lineage dynamics in pancreatic pathology. By integrating 146 samples encompassing more than 300,000 cells, we delineated major pancreatic cell types and demonstrated how their proportions and phenotypes shift with disease progression. As expected from classical pathology, pancreatic cancer exhibited marked expansion of stromal and immune compartments accompanied by loss of acinar cells, whereas fibroblasts and ductal epithelial cells were notably enriched in both primary and metastatic lesions, reflecting the characteristic desmoplastic reaction of pancreatic cancer and the replacement of enzyme-secreting acinar cells by tumorigenic populations.

Single-cell transcriptomic and survival analyses of ductal and acinar cells further highlighted their heterogeneity and clinical relevance. Majority of ductal subtypes were significantly associated with poor patient outcomes, indicating that these transcriptional states have direct clinical implications beyond descriptive classification. However, many acinar subclusters did not confer prognostic impacts, the S100A4⁺ acinar population was consistently linked to adverse clinical outcomes. After integrating the acinar and ductal cell subpopulations, we performed pseudotime analysis to comprehensively investigate the aberrant gene expression patterns underlying the transition from late acinar to early ductal states, ultimately identifying KLF5 and TYMP as two hub molecules. More importantly, we found that the expression levels of KLF5 and TYMP in PRSS1^+^KRT19^+^ cells were substantially higher than those in PRSS1^+^ cells, and exhibited a staircase-like increase, with further elevated expression in KRT19^+^ cells. Transcriptional factor prediction analysis highlighted Klf4 as a potential upstream regulator of Tymp. Moreover, Klf4 exhibited an expression pattern similar to that of Tymp, with both being associated with pancreatic injury and Kras mutation. Collectively, these findings underscore that both ductal and acinar epithelial heterogeneity are critical determinants of pancreatic cancer biology, and they highlight Klf4/Tymp axis as the potential mediator of acinar-to-ductal trajectory that underpins early pancreatic cancer initiation.

KLF4 emerged from our data as a hub gene of acinar cell reprogramming. This finding echoes prior reports that KLF4 induction is critical for ADM [5, 10, 25]. In concordance, we observe that KLF4 upregulation accompanies acinar injury and Kras activation, and overexpression of KLF4 drives loss of acinar markers and gain of ductal markers. This is consistent with KLF4’s known role as a reprogramming factor that can override lineage identity. Lo et al. [26] found that KLF4 overexpression itself suffices to initiate ADM even without Kras mutation, and that chromatin accessibility analysis highlights KLF binding motifs in genes of Kras-downstream pathways (PI3K and Rho GTPases) during ADM. In this study, our results showed that Klf4 promoted the transcriptional level of Tymp and then enhanced Kras-induced ADM processing.

Klf4 and Tymp exhibit highly similar expression patterns. In the reversible ADM model, the levels of Klf4 and Tymp were significantly elevated at days 1–2 post-induction, but returned to baseline by day 14. Furthermore, knockdown of Tymp in the pancreas of KC mice increased the infiltration of CD3⁺ cells, suppressed pancreatic fibrosis, and alleviated the progression of ADM. As a key enzyme involved in nucleotide metabolism, we found that inhibiting Tymp enzymatic activity suppressed ADM development to a certain extent. Enzymatically, Tymp catalyzes the breakdown of thymidine to thymine and 2-deoxy-D-ribose-1-phosphate, a reaction that links nucleotide salvage pathways with cellular energy and redox balance [27, 28]. During ADM, acinar cells re-enter the cell cycle, and upregulation of TYMP could help meet increased demand for nucleotide precursors or mitigate toxic accumulation of thymidine, thereby promoting cell survival during the intense reprogramming phase. Additionally, the 2-deoxy-D-ribose byproduct of TYMP activity has been reported to have pro-angiogenic properties and can modulate the tumor microenvironment [29, 30]. Recent pan-cancer analyses have indeed highlighted TYMP as a gene associated with maintenance of cancer stemness, chemoresistance, and an immunosuppressive microenvironment across multiple tumor types [31]. In the context of ADM, while the cells are not frankly malignant, a TYMP-driven shift could endow emerging neoplastic lesions with a favorable microenvironment (immunosuppressive, pro-angiogenic, pro-growth) early in their development. This mechanism may help explain why areas of ADM create a niche that seemingly favors progression to PanIN and PC. Our study is the first, to our knowledge, to implicate thymidine phosphorylase in the pathogenesis of acinar cell metaplasia and early pancreatic tumorigenesis, thus opening a new avenue for research and intervention.

ADM, the transdifferentiation of pancreatic acinar cells into duct-like cells, is driven by oncogenic KRAS and inflammatory stimuli that co-opt canonical downstream pathways [32, 33]. Genetic models consistently implicate PI3K-AKT signaling as a key driver of ADM. For example, Eser et al. [32] showed that in Kras^G12D^ mice, cell-autonomous PI3K and its effector PDK1 are required for acinar plasticity, ADM and subsequent tumorigenesis. Conversely, transgenic expression of constitutively active PI3Kα (p110α) or AKT1 induces ADM and accelerates PanIN formation, whereas deletion of the p110α subunit blocks injury-induced ADM [34]. Likewise, Costamagna et al. [35] reported that the adaptor p130Cas potentiates KRAS-driven ADM by amplifying PI3K-AKT signaling, and deletion of p130Cas suppresses ADM formation through repression of PI3K-AKT activity. Collectively, these studies suggest that PI3K/AKT signaling promotes the survival, proliferation and cytoskeletal reprogramming needed for ADM initiation.

In parallel, the RAF-MEK-ERK pathway is essential for the ductal conversion itself. In 3D culture of Kras^G12D^-expressing acinar, MEK inhibition completely abrogates ductal cyst formation [33], indicating that MAPK activation is critical for transdifferentiation. Similarly, injury-associated factors such as REG3A/B engage RAS-RAF-MEK-ERK to sustain ADM: Zhang et al. [36] showed that REG3A/REG3B binding to EXTL3 activates RAS-RAF-MEK-ERK and drives persistent ADM and PanIN development. Pharmacologic MEK blockade in pancreatitis models forces acinar redifferentiation and halts metaplasia [37]. In sum, current evidence supports a model in which PI3K/AKT signaling underlies the initiation of ADM and acinar plasticity, whereas sustained MEK/ERK signaling is necessary to maintain ductal reprogramming and enable progression to PanIN. Our study demonstrates that Tymp induces pancreatic ADM by regulating the Pi3k and Mek pathways. While the present study focuses on the role of the Klf4-Tymp axis in inflammation-driven early tumorigenesis, further investigation is warranted to determine whether Tymp also contributes to late-stage pancreatic cancer progression, including metastasis and tumor microenvironment remodeling.

The progression from normal acinar through ADM and PanIN to PC is one of the mainstream theories of pancreatic carcinogenesis, and IPMN is also a well-recognized precancerous lesion of PC. In over 50% of the analyzed IPMN cases, a hotspot mutation was observed in KLF4 gene [38, 39]. Compared with adjacent non-tumor tissues or normal tissues, KLF4 expression was significantly elevated in both low-grade and high-grade IPMN. Moreover, Klf4 overexpression induced pancreatic IPMN-like cystic lesions in the context of Kras mutation [40]. Furthermore, oncogenic activation of the PI3K pathway has also been reported to promote the progression of IPMN [41]. Although the focus of this study is on the ADM process, we also observed that TYMP appears to be associated with IPMN. First, at the transcriptional level, KLF5 and TYMP were identified through cross-dataset analysis including IPMN cohorts, suggesting their potential involvement in IPMN pathogenesis. Additionally, both KLF4 (the upstream transcription factor of TYMP) and the PI3K pathway (a downstream signaling target) have been explicitly reported to promote IPMN development. Nevertheless, whether TYMP regulates IPMN tumorigenesis requires further investigation. Targeting TYMP may also hold potential for suppressing early IPMN initiation.

Our findings identify the KLF4-TYMP axis as a critical driver of inflammation-induced ADM, the earliest initiating event in pancreatic tumorigenesis. While TYMP is not a classical druggable target such as a kinase, its enzymatic activity (catalyzing the reversible phosphorolysis of thymidine) can be pharmacologically inhibited. Clinically approved TYMP inhibitors, such as TPI, which is currently used in combination with trifluridine for metastatic colorectal cancer, offer a potential strategy to target TYMP activity. Although the application of TPI in PC remains to be explored, our results raise the possibility that pharmacological inhibition of TYMP may intercept ADM formation, particularly in high-risk populations with chronic pancreatitis or germline KRAS mutations, where inflammation-driven metaplasia is a prominent feature. Moreover, the upstream regulator KLF4 may serve as a predictive biomarker for identifying individuals with active ADM, enabling early intervention before neoplastic progression. Future studies combining TYMP enzymatic inhibitors with existing anti-inflammatory or chemopreventive strategies may offer a novel approach to reduce the risk of PC initiation. Importantly, because ADM represents a preneoplastic stage, interventions targeting this axis may not require the high intratumoral drug concentrations needed for established tumors, potentially improving tolerability and safety. While direct clinical translation remains speculative at this stage, our findings provide a mechanistic rationale for further exploring the KLF4-TYMP axis as a potential chemoprevention target in high-risk individuals.

## Conclusions

In conclusion, we identify the Klf4-Tymp axis as a critical driver required for KRAS-induced ADM and the initiation of pancreatic tumorigenesis. Mechanistically, Tymp, which is transcriptionally regulated by Klf4, promotes the activation of the Pi3k/Akt and Mek/Erk pathways, ultimately driving pancreatic ADM formation.

## Supplementary Information

Supplementary Material 1

Supplementary Material 2

## Data Availability

The datasets used and/or analysed during the current study are available from the corresponding author on reasonable request.
