# Supplementary material for: Klf4-Tymp axis promotes inflammation-driven early tumorigenesis by enhancing kras mutation-induced acinar-to-ductal metaplasia through Pi3k/Akt and Mek/Erk pathways
Source: J Exp Clin Cancer Res. 2026 May 7;45:177. doi: 10.1186/s13046-026-03712-8 (PMC13474847; doi:10.1186/s13046-026-03712-8)
Supplement: Supplementary file 1 [file 13046_2026_3712_MOESM2_ESM.docx]

**Supplementary Materials**

**Klf4-Tymp Axis Promotes Inflammation-driven Early Tumorigenesis by Enhancing Kras Mutation-Induced Acinar-to-Ductal Metaplasia Through Pi3k/Akt and Mek/Erk Pathways**

**Supplementary Figure 1. Prognostic relevance of ductal subpopulations in pancreatic cancer.** Kaplan-Meier survival curves showing overall survival of patients stratified by the expression of ductal subpopulation-specific gene signatures (D0-D8) based on TCGA datasets. High expression of D0, D1, D3, D4, D5, and D8 ductal signatures was significantly associated with poor prognosis, as indicated by hazard ratios (HR) and log-rank p-values. In contrast, D2, D6, and D7 ductal subpopulations did not exhibit significant prognostic impact.

**Supplementary Figure 2. Prognostic impact of acinar subpopulations in pancreatic cancer.** Kaplan-Meier survival curves showing overall survival of patients stratified by gene signatures specific to acinar subclusters (C0-C4) using TCGA datasets. High expression of the C4 acinar signature was significantly associated with poor prognosis (HR = 1.79, 95% CI: 1.18-2.7, p = 0.005).

**Supplementary Figure 3. Single-cell transcriptomic analysis identifies distinct ductal and acinar cell populations and highlights KLF5 and TYMP expression patterns.** (A) UMAP visualization showing three major cell populations. (B) Dot plot illustrating the expression of canonical ductal markers (KRT19, SOX9) and acinar markers (PRSS1, CPA1) across the three cell populations. (C) Feature plots displaying the distribution of acinar markers PRSS1 and CPA1 across the UMAP plots. (D) Feature plots showing expression of ductal markers KRT19 and SOX9. (E) Feature plots demonstrating the expression patterns of KLF5 and TYMP across cell populations. (F) Quantification of gene expression levels (KLF5, TYMP, PRSS1, and KRT19) across different groups. Statistical significance is indicated as ***P < 0.001.

**Supplementary Figure 4. Clinical significance of TYMP expression in pancreatic cancer.** (A) TYMP expression levels were significantly elevated in tumor tissues compared with adjacent normal tissues across multiple independent cohorts (GSE28735, GSE62452). (B) TYMP expression increased with advancing tumor stage in the TCGA-PAAD dataset. (C) Higher TYMP expression was associated with increased tumor grade across ICGC and TCGA cohorts. (D) Kaplan–Meier survival analyses demonstrated that high TYMP expression consistently correlated with clinical outcomes of patients with PC. (E) qRT-PCR validation showing elevated TYMP mRNA levels in human pancreatic cancer cell lines (PANC1, SW1990) compared with H6C7. (F) Representative immunohistochemistry (IHC) images confirming increased TYMP protein expression in pancreatic cancer tissues relative to para-cancerous tissues. Statistical significance is indicated as *P < 0.05, ***P < 0.001.

**Supplementary Figure 5. Identification and validation of tymp knockdown efficiency.** qRT-PCR results of Tymp expression following shRNA-mediated knockdown in (A) Panc02 cells and (B) primary pancreatic acinar cells. Statistical significance is indicated as *P < 0.05, **P < 0.01, ns, not significant.

**Supplementary Figure 6. Safety evaluation of AAV8-mediated tymp knockdown in vivo.** (A) In vivo bioluminescence imaging confirming efficient pancreas delivery of pAAV8-shRNA(Tymp) at escalating doses compared with control or saline-treated groups. (B) Representative immunofluorescence images of pancreas tissues showing AAV8 transduction efficiency in different treatment groups. (C) Serum biochemical assays, including alanine aminotransferase (ALT), aspartate aminotransferase (AST), albumin (ALB), blood urea nitrogen (BUN), and creatinine (CREA), indicating no significant hepatotoxicity or nephrotoxicity following administration of pAAV8-shRNA(Tymp) across multiple doses. (D) Hematoxylin and eosin (HE) staining of pancreas sections further confirmed preserved pancreatic architecture without evidence of pathological damage in AAV8-shRNA(Tymp)-treated mice.

**Supplementary Figure 7. Impact of AAV8-mediated Tymp knockdown on liver.** Representative immunohistochemistry (IHC) assays confirming no significant difference of tymp expression between AAV-NC and AAV-Tymp groups.

**Supplementary Figure 8. Tymp knockdown suppresses pancreatic ductal remodeling and acinar-to-ductal metaplasia (ADM) in Kras-driven mice.** (A) Representative Alcian Blue staining of pancreatic tissues. (B) Immunofluorescence (IF) staining showing expression of ductal marker CK19 (green), acinar marker amylase (red), and nuclei (DAPI, blue) in pancreatic tissues.

**Supplementary Figure 9. TYMP knockdown inhibits pancreatic cancer cell proliferation, migration and invasion.** (A) Relative TYMP expression levels in cells transfected with control shRNA (shNC) or three independent shRNAs targeting TYMP (shTYMP #1-#3). (B-D) Cell viability assessed by CCK-8 assay at 24h, 48h, and 72h. (E) Wound-healing assay showing cell migration at 0 h and 24 h in shNC and TYMP-knockdown cells. (F) Transwell invasion assay showing decreased invasive capacity in TYMP-knockdown cells compared with control. Statistical significance is indicated as *P < 0.05, **P < 0.01, ***P < 0.001; ns, not significant.

**Supplementary Figure 10. Klf4 directly binds to the Tymp promoter and activates its transcription.** (A) Schematic representation of predicted Klf4-binding motifs within the Tymp promoter region (−2000 to +100 bp relative to the transcription start site, TSS). (B) The dual-luciferase reporter assay verified the regulatory relationship between the transcription factor Klf4 and the Tymp gene. (C) Experimental design for in vivo studies. KC mice were injected intraperitoneally (i.p.) with AAV-Klf4, followed by caerulein treatment. Pancreatic tissues were collected for histological and immunohistochemical analyses at indicated time points. (D) Representative images of HE staining, immunohistochemistry (IHC) for KLF4 and TYMP, and immunofluorescence (IF) staining for amylase (red) and CK19 (green) in Klf4-NC and Klf4-overexpressing (Klf4-OE) mice. (E) Schematic of AAV-mediated Klf4 overexpression in normal mice and subsequent pancreatic organoid derivation. (F) Representative images of pancreatic organoids derived from AAV-Klf4-treated mice with or without thymidine phosphorylase inhibitor (TPI, 20μM). Statistical significance is indicated as ***P < 0.001; ns, not significant.

**Supplementary Figure 11. Tymp promotes pancreatic organoid growth via Pi3k/Akt and Mek/Erk signaling.** (A) Representative bright-field images of pancreatic organoids under different conditions at Days 1, 3, and 5. Compared with control (NC), TYMP overexpression (Tymp-OE) markedly enhances organoid growth, while treatment with the MAPK pathway inhibitor PD98059 (20μM) attenuates this effect. (B) Quantification of organoid relative size at Day 5 in control (NC), Tymp knockdown (Tymp-KD), and Tymp-KD treated with 740 Y-P (20μM). TYMP knockdown significantly reduces organoid growth, which is partially rescued by activation of the PI3K pathway using 740 Y-P. (C) Quantification of organoid relative size at Day 5 in control (NC), Tymp overexpression (Tymp-OE), and Tymp-OE treated with PD98059 (20μM). Statistical significance is indicated as ***P < 0.001.

**Supplementary Figure 12. Conserved TYMP sequence and functional importance of catalytic residues in organoid growth.** Amino acid sequence of (A) human and (B) mouse TYMP with conserved and functionally important residues highlighted. Key catalytic or binding-related residues are marked in red. (C) Molecular docking results of human Tymp protein and thymidine. (D) Molecular docking results of human Tymp protein and thymidine phosphorylase inhibitor (TPI). (E) Representative bright-field images of pancreatic organoids derived from cells expressing control (NC), wild-type TYMP (Tymp-WT), or mutant TYMP (Tymp-Mut) at Days 1, 3, and 5. TYMP overexpression promotes organoid growth, whereas mutation of key residues attenuates this effect. Statistical significance is indicated as ***P < 0.001.
